# Predictors of tolerability for postoperative adjuvant S1 plus docetaxel chemotherapy for gastric cancer: a multicenter retrospective study

**DOI:** 10.1007/s10120-024-01563-w

**Published:** 2024-11-09

**Authors:** Kazuhiro Toyota, Kazuaki Tanabe, Mikihiro Kano, Toshiaki Komo, Ryuichi Hotta, Senichiro Yanagawa, Yoshihiro Saeki, Hirofumi Tazawa, Masahiro Ikeda, Masayuki Shishida, Keisuke Okano, Ryuta Ide, Yasuhiro Imaoka, Shinya Takahashi, Hideki Ohdan, the Hiroshima Surgical Study Group of Clinical Oncology (HiSCO), Nobuaki Fujikuni, Toshihiro Misumi, Jun Hihara, Noriaki Tokumoto, Yoichi Sugiyama, Yuji Yamamoto, Takahisa Suzuki, Yoshihiro Sakashita, Toshikatsu Fukuda

**Affiliations:** 1 Department of Surgery, Hiroshima Memorial Hospital, 1-4-3, Honkawa-cho, Naka-ku, Hiroshima, Hiroshima 730-0802 Japan; 2 Department of Perioperative and Critical Care Management, Graduate School of Biomedical and Health Sciences, Hiroshima University, 1-2-3, Kasumi, Minami-ku, Hiroshima, 734-8551 Japan; 3 Department of Surgery, Hiroshima City North Medical Center Asa Citizens Hospital, Hiroshima, Japan; 4 Department of Surgery, JA Hiroshima General Hospital, Hatsukaichi, Japan; 5 Department of Surgery, National Hospital Organization Higashihiroshima Medical Center, Higashihiroshima, Japan; 6 Department of Surgery, JA Onomichi General Hospital, Onomichi, Japan; 7 Department of Gastroenterological and Transplant Surgery, Graduate School of Biomedical and Health Sciences, Hiroshima University, Hiroshima, Japan; 8 Department of Surgery, Kure Medical Center, Chugoku Cancer Center, Kure, Japan; 9 Department of Surgery, Chuden Hospital, Hiroshima, Japan; 10 Department of Surgery, JR Hiroshima Hospital, Hiroshima, Japan; 11 Department of Surgery, Miyoshi Central Hospital, Miyoshi, Japan; 12 Department of Surgery, Chugoku Rosai Hospital, Kure, Japan; 13 Department of Surgery, JA Yoshida General Hospital, Akitakata, Japan; 14 Department of Surgery, Graduate School of Biomedical and Health Science, Hiroshima University, Hiroshima, Japan

**Keywords:** Docetaxel, S1, Gastric cancer, Adjuvant chemotherapy, Tolerability

## Abstract

**Background:**

Adjuvant docetaxel plus S1 (DS) chemotherapy after gastrectomy with D2 lymph node dissection is the standard treatment for stage III gastric cancer in Japan; however, some patients are unable to receive adequate drug administration because of the deterioration of their conditions. This study aimed to investigate the correlation between tolerability for postoperative adjuvant DS chemotherapy and prognosis, and the factors affecting tolerability.

**Methods:**

This retrospective study involved patients with stage III gastric cancer who underwent curative resection between 2018 and 2021 from a multicenter database. Patients with a cumulative dose of docetaxel and S1 greater than 120 and 8400 mg/m^2^, respectively, were considered tolerable. The prognostic impact and factors predicting tolerability were analyzed.

**Results:**

Of the 103 patients, the tolerable group comprised of 63 (61%) patients and had a significantly better 3-year recurrence-free survival than the intolerable group (83% vs. 64%, P = 0.02). Among the preoperative factors, only performance status (PS, P = 0.04) was significantly correlated with tolerability in the univariate analysis. Among the postoperative factors, PS (P = 0.001) and perioperative weight loss rate (P = 0.02) were significantly correlated with tolerability in the univariate analysis. The multivariate analysis showed significant differences in the PS (odds ratio [OR]: 4.94, 95% confidence interval [CI] 1.79–14.98, P = 0.002) and weight loss rate (OR: 1.10, 95% CI 1.01–1.21, P = 0.03).

**Conclusions:**

Tolerance to postoperative adjuvant DS chemotherapy has a significant prognostic impact. Postoperative PS and perioperative weight loss rates were independent predictors of tolerability.

## Introduction

Although the incidence of gastric cancer is declining, it remains the fifth leading cause of cancer-related deaths worldwide, accounting for approximately 660,000 deaths annually [1]. The European Society for Medical Oncology (ESMO) guidelines recommend surgery and perioperative chemotherapy for locally advanced gastric cancer [2].

In Japan, gastrectomy with D2 lymph node dissection and adjuvant chemotherapy are the standard of care [3]. In patients with stage III gastric cancer, postoperative docetaxel plus S1 (DS) chemotherapy was shown to improve recurrence-free survival (RFS) and overall survival (OS) in the JACCRO GC-07 trial, and is one of the recommended treatments in the Japanese gastric cancer guidelines [4–6].

Patients who undergo gastrectomy often have decreased oral intake, and deterioration of their physical and nutritional conditions may affect their ability to tolerate postoperative chemotherapy. In the JACCRO GC-07 trial, patients with postoperative gastric cancer received six courses of docetaxel every 3 weeks and S1 for 1 year, with completion rates of 71% and 49% for each drug, respectively. Improvement in tolerability requires investigation of the factors that influence the lower completion rate and the development of a better care protocol from the start of chemotherapy.

Factors reported to influence the intolerance for adjuvant chemotherapy for gastric cancer include postoperative weight loss, older age, and infectious complications [7, 8]. However, these have been reported for postoperative S1 monotherapy, and there are no reports on DS chemotherapy, which requires more intensive management to maintain compliance. We hypothesized that elucidating the predictors for DS therapy tolerability would allow appropriate measures to be taken and potentially improve patient prognosis. We designed a study using a multicenter database to search for factors that influence intolerance among a number of factors. This study aimed to elucidate the relationship between the tolerability and prognosis of postoperative DS chemotherapy for gastric cancer, and the factors that influence tolerability.

## Materials and methods

### Patients

We reviewed multicenter cases registered in the Hiroshima Surgical Study Group of the Clinical Oncology (HiSCO) Gastric Cancer database. The inclusion criteria were as follows: patients who underwent curative (R0) surgery for gastric cancer between 2018 and 2021; patients who were diagnosed with pathological stage III disease according to the 15th edition of the Japanese Classification of Gastric Carcinoma, which was identical to the 8th edition of the TNM classification [9]; and patients who received DS adjuvant chemotherapy postoperatively. To assess the tolerability to DS therapy, patients who completed DS chemotherapy or discontinued it due to adverse events were included; patients who discontinued the treatment for other reasons, such as relapse, were excluded. All procedures were in accordance with the principles of the Declaration of Helsinki and Ethical Guidelines for Medical and Health Research Involving Human Subjects. The study protocol was approved by the Institutional Review Board of Hiroshima Memorial Hospital (approval number: 23061901). This study was a part of the Hiroshima Surgical Study Group of Clinical Oncology (HiSCO) database, and informed consent was obtained from patients in the form of an opt-out on the website of each institution.

### Procedures

Postoperative DS adjuvant chemotherapy was administered following previous reports [4]. The dosing for S1 was determined according to body surface area (< 1.25 m^2^, 80 mg/day; 1.25–1.5 m^2^, 100 mg/day; and ≥ 1.5 m^2^, 120 mg/day) and divided into two doses per day. During the first course, the patients received S1 orally from days 1–14 of the 3-week course. During the second to seventh courses, the patients received an intravenous infusion of docetaxel (40 mg/m^2^ body surface area) on day 1 and S1 orally on days 1–14 of the 3-week course. After the eighth course, S1 was taken on days 1–28 of the 6-week course and continued until 1 year postoperatively. Chemotherapy was initiated as soon as possible after surgery and continued for as long as possible by reducing the dose according to the degree of adverse events. Adverse events were monitored by evaluating clinical symptoms and blood tests at least once in each course and graded according to the Common Terminology Criteria for Adverse Events version 5.0. Patients with uncontrolled adverse events or gastric cancer recurrence were terminated. Recurrence was evaluated based on clinical and imaging findings, with standard computed tomography every 6 months and gastrointestinal endoscopy every 12 months.

### Evaluation

Clinical findings and blood test results were obtained from medical records, and the relationships between physical status, blood cell counts, organ function, nutritional status, inflammatory status, and tolerability for DS chemotherapy were analyzed. The following indicators of liver function, renal function, nutritional status, and inflammatory status were used: albumin-to-bilirubin index = log_10_ (total bilirubin [mg/dL] × 17.1) × 0.66 + Albumin [g/L] × 10 × − 0.085); estimated glomerular filtration rate; lymphocyte-to-monocyte ratio (LMR) = total lymphocyte count (TLC)/monocyte count; neutrophil-to-lymphocyte ratio = neutrophil count/TLC; platelet-to-lymphocyte ratio = TLC/platelet count × 100; platelet-to-neutrophil ratio = neutrophil count/platelet count × 100), Onodera’s prognostic nutritional index (PNI) = 10 × Albumin [g/L] + 0.005 × TLC; controlling nutrition status score, which was calculated using the albumin, TLC, and cholesterol levels; Glasgow prognostic score (GPS), which was calculated using the C-reactive protein (CRP) [mg/dL] and albumin; CRP-albumin ratio = CRP [mg/dL]/albumin [g/L]); and systemic inflammation score, which was calculated using the albumin and LMR [10, 11]. Preoperative and postoperative data (before the initiation of DS chemotherapy), and data at the end of second course of DS chemotherapy were analyzed.

### Statistical analysis

Tolerability to DS chemotherapy was classified using cumulative doses of both drugs: patients with cumulative doses of docetaxel ≥ 120 mg/m^2^ and S1 ≥ 8400 mg/m^2^ were in the tolerable group, and patients not included in this group were classified as the intolerable group. RFS was defined as the period from the date of surgery to recurrence. Survival curves were generated using the Kaplan–Meier method and compared using the log-rank test. Odds ratios (OR) were calculated from the relationship between the two groups and each factor using logistic regression analysis. Multivariate analysis was performed using multiple logistic regression analysis for factors that showed significant differences in the univariate analysis to analyze the independent predictors of tolerability. Statistical significance was set at P < 0.05. Statistical analyses were performed using JMP software version 12 (SAS Institute, Cary, NC, USA).

## Results

**Fig. 1:**
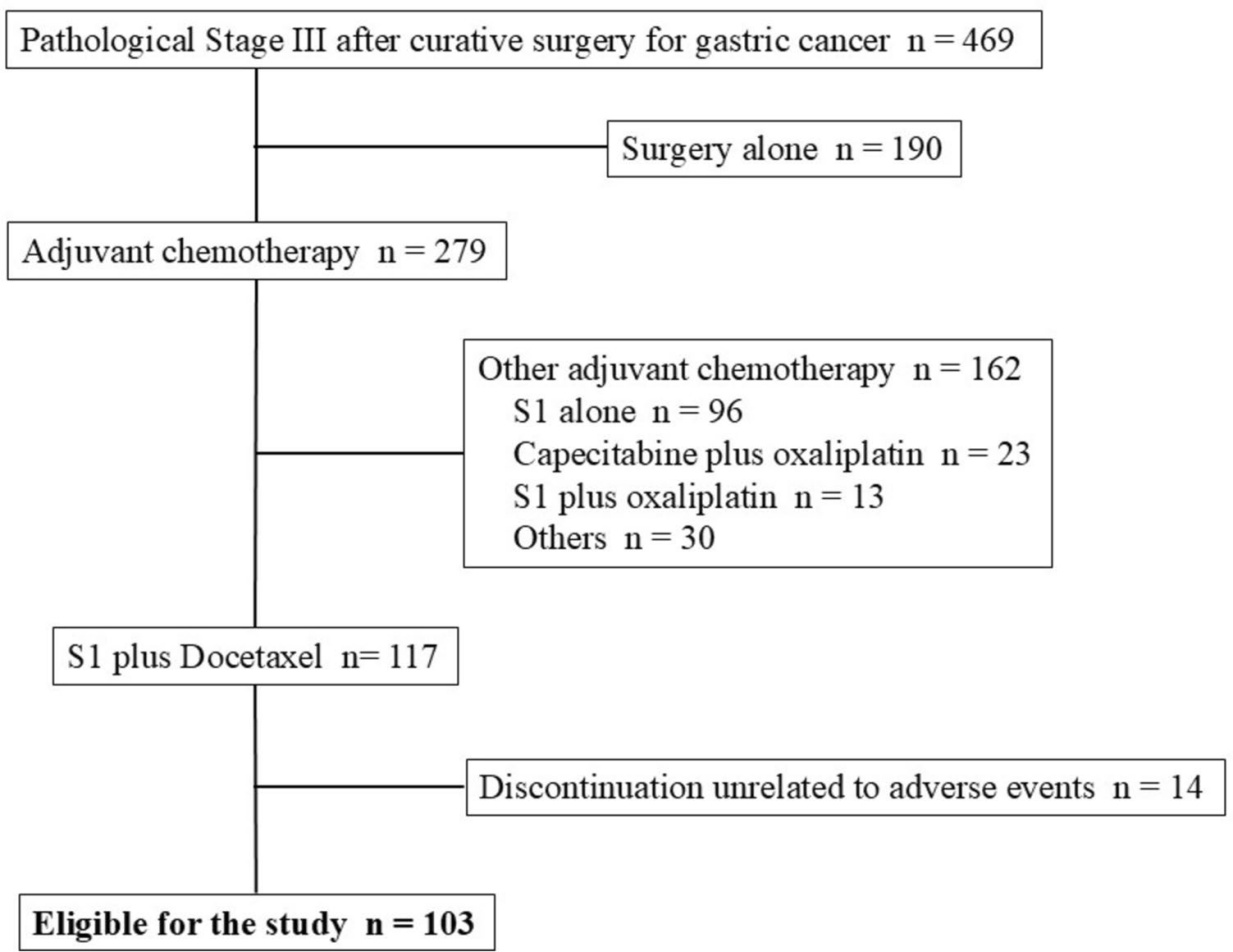
Flow diagram of patient selection

Of the 2574 patients who underwent surgery for gastric cancer between 2018 and 2021 in the HiSCO database, 469 underwent curative (R0) surgery and were diagnosed with pathological stage III gastric cancer. Postoperative DS therapy was performed in 117 patients. Of the patients who discontinued treatment the following 14 patients were excluded from the study because they were not considered to be affected by adverse events: 11 patients who discontinued treatment due to recurrence of gastric cancer, two patients with bowel obstruction surgery, one patient who was lost to follow-up. Finally, 103 patients who either completed postoperative DS therapy or discontinued it due to adverse events were eligible for the study (Fig. 1). The patient characteristics are shown in Table 1. The age of the patients ranged from 26 to 81 years (median, 70 years), and 71 patients (69%) were male and 32 (31%) were female. Six patients (6%) with oesophagogastric junction cancer were included. No patients with remnant gastric cancer were included. Six (6%) patients who received preoperative chemotherapy were included. A total of 65 patients (63%) underwent distal gastrectomy and 38 (37%) underwent total gastrectomy. The number of patients with pathological stages IIIA, IIIB, and IIIC were 61 (59%), 26 (25%), and 16 (16%), respectively. The median postoperative follow-up period was 33.6 months.

**Table 1:** Patient characteristics (n = 103)

Variables	Patients (%)
Age	
Years (range)	70 (26–81)
Gender	
Male	71 (69)
Female	32 (31)
Preoperative body mass index	
Mean ± SD	22.7 ± 3.6
Preoperative comorbidities	
Absent	31 (30)
Present	72 (70)
Preoperative performance status	
0	89 (86)
1	12 (12)
2	2 (2)
ASA physical status	
1	14 (14)
2	84 (81)
3	5 (5)
Preoperative chemotherapy	
Not performed	97 (94)
Performed	6 (6)
Type of gastrectomy	
Distal	65 (63)
Total	38 (37)
Type of surgical approach	
Open	70 (68)
Laparoscopic	31 (30)
Robotic	2 (2)
Lymph node dissection	
D1+	5 (5)
D2	98 (95)
Combined resection of other organs	
Not performed	57 (55)
Performed	46 (45)
Tumor differentiation	
Differentiated	41 (40)
Undifferentiated	62 (60)
Pathological T factor	
T2,3	47 (46)
T4	56 (54)
Pathological N factor	
N0–2	61 (59)
N3	42 (41)
Pathological stage	
IIIA	61 (59)
IIIB	26 (25)
IIIC	16 (16)

*SD* standard deviation, *ASA* American Society of Anesthesiologists

The incidence of adverse events associated with DS chemotherapy is shown in Table 2. The common adverse events were leukopenia, neutropenia, anorexia, diarrhea, fatigue, and alopecia. Of the total, 52 (50%) patients experienced grade 3 or higher adverse events. No patients died of adverse events (grade 5). Our results regarding adverse events were similar to those of previous reports [4], except that there were fewer grade 1–2 increases in the aspartate aminotransferase and alanine aminotransaminase.

**Table 2:** Adverse events by grade

	All grades (%)	Grade 3/4 (%)
Leukopenia	39 (38)	16 (16)
Neutropenia	49 (48)	37 (36)
Febrile neutropenia	7 (7)	7 (7)
Anemia	5 (5)	1 (1)
Thrombocytopenia	3 (3)	0 (0)
AST increased	3 (3)	2 (2)
ALT increased	3 (3)	2 (2)
Bilirubin increased	3 (3)	1 (1)
Creatinine increased	1 (1)	0 (0)
Anorexia	62 (60)	9 (9)
Nausea	21 (20)	1 (1)
Vomiting	4 (4)	0 (0)
Diarrhea	30 (29)	5 (5)
Mucositis oral	12 (12)	1 (1)
Fatigue	26 (25)	2 (2)
Alopecia	42 (41)	–

Adverse event grades were determined using the Common Terminology Criteria for Adverse Events version 5.0

*AST* aspartate transferase, *ALT* alanine transaminase

Median (interquartile range) cumulative doses of docetaxel and S1 were 184 (70–217) mg/m^2^ and 11,657 (6442–15,004) mg/m^2^, respectively. A total of 63 patients (61%) were classified into the tolerable group and 40 (39%) into the intolerable group.

**Fig. 2:**
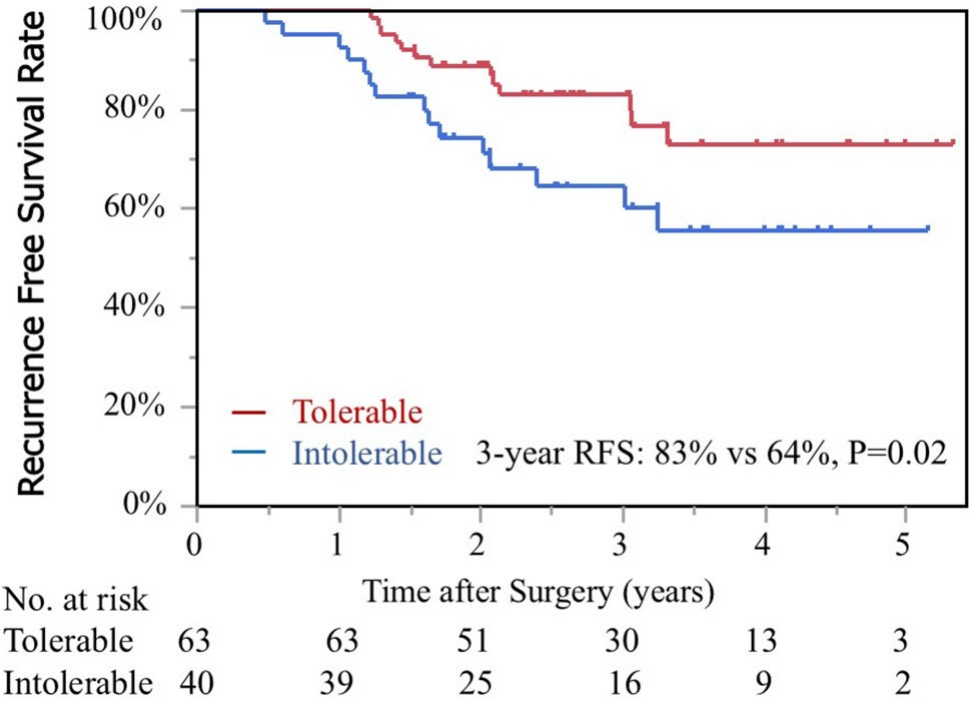
RFS curve stratified by the tolerability for postoperative S1 plus docetaxel chemotherapy. *RFS* recurrence-free survival

A total of 27 patients (26%) experienced recurrence after DS chemotherapy. The recurrence sites included the peritoneum (n = 15), lymph nodes (n = 6), bone (n = 2), liver (n = 1), lung (n = 1), and others (n = 5). The 3-year RFS rates for the tolerable and intolerable groups were 83% and 64%, respectively, and the tolerable group showed a significantly better RFS (P = 0.02, Fig. 2). Only nine deaths occurred during the observation period, including deaths from other diseases.

Table 3 shows the results of the analysis on the impact of preoperative factors on the tolerability of postoperative DS chemotherapy. The univariate analysis showed significant differences in the preoperative Eastern Cooperative Oncology Group Performance Status (PS, OR: 3.37, 95% confidence interval [CI]: 1.04–10.93, P = 0.04).

**Table 3:** Analysis of preoperative factors affecting tolerability

Variables	Tolerable	Intolerable	Univariate analysis			Multivariate analysis		
	Group (n = 63)	Group (n = 40)	OR	95% CI	*p* value	OR	95% CI	*p* value
Age, median (IQR)	69 (64–74)	72 (66–78)	0.97	0.92–1.01	0.17			
Gender, male	42 (67%)	29 (73%)	1.32	0.55–3.14	0.53			
Body mass index, mean ± SD	22.9 ± 3.8	22.5 ± 3.2	1.04	0.93–1.16	0.54			
Presence of symptoms	40 (63%)	29 (73%)	1.52	0.64–3.59	0.34			
Presence of comorbidity	45 (71%)	27 (68%)	0.83	0.35–1.96	0.67			
Preoperative chemotherapy	5 (8%)	1 (3%)	0.30	0.03–2.64	0.25			
Performance status of 1 or 2	5 (8%)	9 (23%)	3.37	1.04–10.93	0.04			
ASA physical status of 2 or 3	53 (84%)	36 (90%)	1.70	0.49–5.84	0.40			
Total bilirubin, median (IQR)	0.5 (0.4–0.8)	0.5 (0.4–0.7)	2.09	0.53–9.35	0.30			
Albumin-to-bilirubin index, median (IQR)	-2.65 (-2.79–-2.41)	-2.54 (-2.83–-2.17)	0.80	0.34–1.89	0.61			
Cholinesterase, median (IQR)	289 (225–345)	260 (199–318)	1.00	0.99–1.01	0.16			
Creatinine, median (IQR)	0.80 (0.67–0.95)	0.81 (0.68–0.99)	0.50	0.07–3.34	0.47			
Estimated glomerular filtration rate, median (IQR)	71.0 (61.4–80.0)	69.1 (59.1–79.3)	0.99	0.97–1.02	0.86			
White blood cell, median (IQR)	6360 (5310–7310)	6690 (5265–8065)	0.99	0.99–1.00	0.15			
Hemoglobin, median (IQR)	13.1 (11.5–14.6)	12.9 (10.7–13.9)	1.10	0.92–1.33	0.28			
Platelet (× 10^4^), median (IQR)	24.4 (20.9–30.7)	27.3 (21.9–35.2)	0.98	0.93–1.02	0.30			
Lymphocyte-to-monocyte ratio, median (IQR)	4.51 (3.29–5.74)	3.98 (3.00– 5.58)	1.11	0.90–1.39	0.34			
Neutrophil-to-lymphocyte ratio, median (IQR)	2.26 (1.62–3.08)	2.53 (1.99–3.80)	0.84	0.62–1.11	0.23			
Platelet-to-lymphocyte ratio, median (IQR)	143 (112–203)	160 (136–225)	0.99	0.99–1.00	0.72			
Platelet-to-neutrophil ratio, median (IQR)	1.58 (1.15–1.90)	1.66 (1.24–2.06)	0.69	0.32–1.46	0.33			
C-reacted protein, median (IQR)	0.13 (0.05–0.27)	0.15 (0.05–0.64)	0.98	0.77–1.27	0.84			
Total protein, mean ± SD	6.8 ± 0.7	6.7 ± 0.6	1.21	0.67–2.20	0.52			
Albumin, mean ± SD	3.9 ± 0.6	3.8 ± 0.6	1.33	0.67–2.65	0.42			
Total cholesterol, mean ± SD	189 ± 38	182 ± 43	1.00	0.99–1.02	0.41			
Onodera’s prognostic nutritional index, mean ± SD	47.4 ± 7.2	46.3 ± 6.2	1.02	0.97–1.09	0.42			
Controlling Nutrition Status score of 2–12	19 (35%)	15 (45%)	1.54	0.63–3.72	0.34			
Glasgow prognostic score of 1 or 2	11 (19%)	12 (35%)	2.38	0.91–6.22	0.07			
C-reactive protein—albumin ratio, median (IQR)	0.032 (0.011–0.067)	0.043 (0.012–0.158)	0.98	0.51–2.05	0.94			
Systemic inflammation score of 1 or 2	48 (79%)	32 (80%)	1.08	0.40–2.91	0.87			
Carcinoembryonic antigen, median (IQR)	2.3 (1.3–4.3)	2.1 (1.4–3.3)	1.06	0.99–1.25	0.11			
Carbohydrate antigen 19–9, median (IQR)	7.3 (3.7–14.2)	6.0 (2.3–16.3)	1.00	0.99–1.01	0.22			

OR, odds ratio; CI, confidence interval; IQR, interquartile range; SD, standard deviation; ASA, American Society of Anesthesiologists

The results of the analysis of the association between postoperative factors and tolerability are presented in Table 4. The univariate analysis showed significant differences in the perioperative weight loss rate (OR: 1.10, 95% CI 1.01–1.21, P = 0.02) and postoperative PS (OR: 4.80, 95% CI 1.74–13.2, P = 0.001). The multivariate analysis showed that high perioperative weight loss rate (OR: 1.10, 95% CI 1.01–1.21, P = 0.03) and poor postoperative PS (OR: 4.94, 95% CI 1.79–14.98, P = 0.002) were independent predictors of poor tolerability for postoperative DS chemotherapy.

**Table 4:** Analysis of postoperative factors affecting tolerability

Variables	Tolerable	Intolerable	Univariate analysis			Multivariate analysis		
	Group (n = 63)	Group (n = 40)	OR	95% CI	*p* value	OR	95% CI	*p* value
Type of gastrectomy, total	20 (32%)	18 (45%)	1.76	0.78–3.99	0.17			
Lymph node dissection, D2	60 (95%)	38 (95%)	0.95	0.15–5.95	0.96			
Open surgical approach	43 (68%)	27 (68%)	0.97	0.41–2.25	0.94			
Performed combined resection	25 (40%)	21 (53%)	1.68	0.75–3.74	0.20			
Operative time (minutes), median (IQR)	291 (256–325)	296 (230–312)	1.00	0.99–1.01	0.14			
Intraoperative bleeding (mL), median (IQR)	150 (50–220)	103 (50–270)	0.99	0.99–1.00	0.48			
Postoperative complications of grade 2 or 3^a^	14 (22%)	6 (15%)	0.62	0.22–1.77	0.37			
Tumor differentiation, undifferentiated	39 (62%)	23 (58%)	0.83	0.37–1.87	0.66			
T4 pathological factor	37 (59%)	19 (48%)	0.64	0.29–1.41	0.26			
N3 pathological factor	21 (33%)	21 (53%)	2.21	0.98–4.98	0.05			
Pathological stage of IIIB or IIIC	22 (35%)	20 (50%)	1.86	0.83–4.18	0.13			
Body mass index, mean ± SD	21.5 ± 3.4	20.6 ± 3.1	1.09	0.96–1.25	0.17			
Perioperative body weight loss rate (%), median (IQR)	6.46 (3.51–9.33)	7.65 (4.64–11.99)	1.10	1.01–1.21	0.02	1.10	1.01–1.21	0.03
Performance status of 1	7 (11%)	15 (38%)	4.80	1.74–13.2	0.001	4.94	1.79–14.98	0.002
Total bilirubin, median (IQR)	0.5 (0.4–0.7)	0.5 (0.4–0.7)	0.62	0.12–3.35	0.58			
Albumin-to-bilirubin index, median (IQR)	-2.64 (-2.81–-2.48)	-2.66 (-2.82–-2.42)	0.67	0.18–2.40	0.53			
Creatinine, median (IQR)	0.74 (0.62–0.90)	0.78 (0.65–0.95)	0.51	0.07–3.72	0.5			
Estimated glomerular filtration rate, median (IQR)	73.2 (63.3–80.3)	69.2 (59.7–81.5)	1.00	0.98–1.03	0.55			
White blood cell, median (IQR)	5800 (4720–6990)	5095 (4418–6313)	1.00	0.99–1.00	0.29			
Hemoglobin, median (IQR)	12.3 (11.0–13.3)	12.1 (10.7–12.8)	1.13	0.85–1.52	0.41			
Platelet (× 10^4^), median (IQR)	24.8 (20.8–30.2)	23.2 (20.5–29.9)	1.01	0.96–1.08	0.63			
Lymphocyte-to-monocyte ratio, median (IQR)	5.00 (3.82–5.93)	4.35 (3.13–6.29)	1.03	0.84–1.29	0.75			
Neutrophil-to-lymphocyte ratio, median (IQR)	1.78 (1.41–2.27)	2.19 (1.58–2.50)	0.91	0.59–1.39	0.65			
Platelet-to-lymphocyte ratio, median (IQR)	143 (95–196)	161 (120–202)	0.99	0.99–1.00	0.49			
Platelet-to-neutrophil ratio, median (IQR)	1.23 (0.93–1.90)	1.25 (0.94–1.57)	1.40	0.69–2.99	0.36			
C-reactive protein, median (IQR)	0.08 (0.03–0.19)	0.10 (0.04–0.55)	0.77	0.36–1.56	0.45			
Total protein, mean ± SD	6.7 ± 0.5	6.7 ± 0.5	1.36	0.58–3.31	0.49			
Albumin, mean ± SD	3.8 ± 0.3	3.8 ± 0.4	1.25	0.44–3.60	0.67			
Onodera’s prognostic nutritional index, mean ± SD	47.4 ± 4.4	46.2 ± 5.4	1.05	0.97–1.15	0.21			
Glasgow prognostic score of 1 or 2	8 (15%)	5 (15%)	0.97	0.29–3.27	0.97			
C-reactive protein—albumin ratio, median (IQR)	0.022 (0.007–0.047)	0.032 (0.011–0.139)	0.42	0.03–4.94	0.47			
Systemic inflammation Score of 1 or 2	42 (67%)	32 (80%)	2.00	0.79–5.10	0.14			
Carcinoembryonic antigen, median (IQR)	2.1 (1.2–2.9)	1.9 (1.5–2.9)	1.04	0.89–1.27	0.64			
Carbohydrate antigen 19–9, median (IQR)	5.2 (3.0–9.2)	6.0 (2.0–11.0)	1.01	0.99–1.06	0.13			

*OR* odds ratio, *CI* confidence interval

^a^Clavien-Dindo classification

Table 5 shows the result of the analysis of factors affecting tolerability at the end of second courses of DS chemotherapy. Ten patients discontinued due to adverse events by end of two course of DS chemotherapy. Data were not available for one patient. In univariate analysis, the only factor that significantly affected tolerability was PS (OR: 7.71, 95% CI 2.74–21.66, P < 0.001).

**Table 5:** Analysis of factors at the end of second courses of S-1 plus docetaxel therapy affecting tolerability

Variables	Tolerable	Intolerable	Univariate analysis			Multivariate analysis		
	Group (n = 62)	Group (n = 30)	OR	95% CI	*p* value	OR	95% CI	*p* value
Age, median (IQR)	69 (64–74)	71 (66–76)	0.96	0.90–1.02	0.18			
Gender, male	41 (66%)	23 (77%)	1.68	0.62–4.56	0.30			
Body mass index, median (IQR)	20.0 (18.7–23.7)	20.1 (18.4–22.2)	1.09	0.94–1.28	0.25			
Body weight loss rate since pre-surgery (%), median (IQR)	8.14 (4.80–11.95)	9.69 (5.42–13.96)	1.04	0.98–1.12	0.24			
Body weight loss rate since start of DS therapy (%), median (IQR)	1.73 (0.00–5.00)	2.05 (-0.16–3.72)	0.99	0.89–1.11	0.92			
Performance status of 1 or 2	8 (13%)	16 (53%)	7.71	2.74–21.66	< 0.001			
Total bilirubin, median (IQR)	0.6 (0.5–0.9)	0.7 (0.5–0.9)	0.58	0.16–2.07	0.40			
Albumin-to-bilirubin index, median (IQR)	-2.38 (-2.57–-2.15)	-2.36 (-2.66–-2.07)	1.02	0.42–2.81	0.96			
Creatinine, median (IQR)	0.72 (0.63–0.83)	0.80 (0.67–0.90)	0.32	0.03–3.74	0.36			
Estimated glomerular filtration rate, median (IQR)	78.2 (68.5–90.5)	76.3 (65.6–88.1)	1.01	0.98–1.04	0.49			
White blood cell, median (IQR)	5350 (4380–7323)	6150 (4100–6858)	1.00	0.99–1.00	0.35			
Hemoglobin, median (IQR)	11.4 (10.9–12.2)	10.8 (10.2–12.2)	1.19	0.81–1.76	0.37			
Platelet (× 10^4^), median (IQR)	25.9 (20.8–30.3)	26.5 (19.8–34.1)	1.00	0.95–1.06	0.91			
Lymphocyte-to-monocyte ratio, median (IQR)	3.83 (2.95–5.65)	3.62 (2.25–5.58)	1.06	0.93–1.28	0.41			
Neutrophil-to-lymphocyte ratio, median (IQR)	1.64 (1.28–2.31)	1.88 (1.50–2.66)	0.86	0.53–1.38	0.52			
Platelet-to-lymphocyte ratio, median (IQR)	146.3 (99.6–190.9)	157.0 (109.5–196.4)	0.99	0.99–1.00	0.64			
Platelet-to-neutrophil ratio, median (IQR)	1.23 (0.82–1.70)	1.20 (0.95–1.55)	1.22	0.65–2.57	0.54			
Total protein, mean ± SD	6.5 ± 0.4	6.5 ± 0.46	1.06	0.39–2.94	0.91			
Albumin, mean ± SD	3.8 ± 0.4	3.8 ± 0.4	1.26	0.40–4.05	0.70			
Onodera’s prognostic nutritional index, mean ± SD	40.4 ± 3.6	40.3 ± 4.7	1.00	0.90–1.12	0.95			
Glasgow prognostic score of 1 or 2	11 (23%)	7 (32%)	1.57	0.51–4.82	0.43			
Systemic inflammation Score of 1 or 2	56 (92%)	27 (90%)	0.80	0.18–3.61	0.78			
Carcinoembryonic antigen, median (IQR)	3.3 (2.1–4.9)	3.5 (2.5–4.6)	1.09	0.90–1.37	0.38			
Carbohydrate antigen 19–9, median (IQR)	6.6 (3.0–12.2)	4.0 (2.3–9.2)	1.06	0.99–1.15	0.10			

## Discussion

This study showed that the cumulative dose of postoperative DS chemotherapy for stage III gastric cancer affects RFS and that postoperative PS and perioperative body weight loss rate are independent predictors of tolerability. To our knowledge, there have been no reports on the predictors of tolerability for postoperative DS chemotherapy. We aimed to elucidate on this by analyzing several parameters in a multicenter study.

Gastrectomy with D2 lymph node dissection is performed as a curative surgery for gastric cancer in Japan. Adjuvant therapy has been developed to improve the survival of patients with advanced gastric cancer. The addition of S1 chemotherapy after surgery in patients with stage II and III gastric cancer has been shown to improve OS better than surgery alone [12]. For patients with stage III gastric cancer, S1 alone was considered inadequate, and the addition of docetaxel was shown to be effective [4]. Postoperative DS therapy is considered one of the standard treatments for stage III gastric cancer according to the Japanese guidelines [3]. Adequate administration of both drugs is necessary. In this study, postoperative PS and weight regain were found to be significant. Although further development of effective postoperative treatments is expected, more aggressive postoperative chemotherapy is burdensome and difficult due to the deterioration of the physical and nutritional conditions of patients after gastrectomy. In this study, DS therapy was indicated for patients with PS 0 or 1, as in the JACCRO-GC-07 study (Table 4). Nevertheless, 40 patients (39%) were classified as intolerant. In Europe, the efficacy of perioperative chemotherapy has been reported [13–15] and is recommended in the ESMO guidelines [2]. In Japan, additional pre-operative chemotherapeutic regimens have been developed and tested in clinical trials. The maintenance and improvement of PS and weight in the perioperative period in patients with gastric cancer may become more important in the future.

In previous reports, the duration of administration and cumulative dose were used as indicators for chemotherapy tolerability, and studies on factors affecting the tolerability for postoperative S1 chemotherapy used the duration of administration as an indicator [8, 10, 11, 16]. There are several problems with the duration of administration as a measure of tolerability for DS chemotherapy. S1 and docetaxel have different durations of administration (1 year for S1 and 6 months for docetaxel), and categorizing each separately would yield less statistical power and would be complicated. Simply dividing the patients into two groups based on the duration of chemotherapy was also problematic because the discontinuation of one drug did not affect the analysis. This was because patients who received one drug for a long period and the other for only a short period were considered the same as patients who received both drugs sufficiently. Because of these difficulties in using the duration of administration as an indicator, the cumulative dose was used as an indicator for tolerability. Because the addition of docetaxel to S1 has been shown to improve prognosis [4], elucidating the factors that might allow the administration of both drugs rather than one or the other was considered to be important. Therefore, we classified the patients into two groups: those who received sufficient doses of both drugs and those who did not. The dose cutoffs were set at cumulative doses of 8400 mg/m^2^ for S1 and 120 mg/m^2^ for docetaxel. The results showed a significant difference in the RFS between the two groups. Therefore, we find this classification method to be legitimate and useful.

Although the effectiveness of postoperative adjuvant chemotherapy in patients with gastric cancer has been demonstrated [4, 12], in practice, some patients are unable to receive adequate doses. The need to clarify the factors affecting tolerability has been recognized. The effects of older age, postoperative infectious complications, PNI, and postoperative weight loss on the tolerability for postoperative S1 adjuvant chemotherapy for gastric cancer have been previously reported [7, 8, 16]. These results suggest that the postoperative physical condition and nutritional status of patients affect the tolerability for chemotherapy, which may represent the characteristics of post-gastrectomy patients who are prone to these declines. Poor S1 efficacy owing to weight loss also leads to poor survival in gastric cancer [17]. In the current study, we evaluated only postoperative DS chemotherapy with poorer compliance than S1 monotherapy and analyzed several factors related to the physical condition, degree of surgical invasion, inflammatory system markers, and nutritional status of patients. Among the many factors analyzed, the influence of PS and weight loss was particularly significant. In the univariate analysis at the end of second courses of DS chemotherapy, PS was the only factor that showed a significant difference. The smaller number of intolerant group of patients may have weakened the statistical difference. Because not a few patients discontinued treatment before the end of the second course, tolerability evaluation before chemotherapy may be useful. We hope that attention to these factors will enable appropriate care, condition assessment, chemotherapeutic drug selection, and timing decisions regarding chemotherapy initiation in patients with gastric cancer.

The current study had several limitations. This was a retrospective, multi-institutional study, and there may have been some differences in treatment protocols at each institution. However, all participating institutions treated the patients according to the Japanese Gastric Cancer Treatment Guidelines [3, 18], and the adverse events and completion rates of DS chemotherapy were similar to previous reports [4]. We believe that differences between institutions did not significantly affect the results of the current study. Selection bias was evident in the study. Only patients selected for postoperative DS chemotherapy were included in this study, and many patients were excluded from the study because they did not receive postoperative chemotherapy or S1 alone. Only patients who were expected to be tolerable for DS chemotherapy may have been selected. The follow-up period of the study was short (median, 33.6 months). It is possible that some patients will relapse in the future, which may affect the results of the RFS analysis. Due to the low incidence of death, OS was not able to analyze. However, not enough time has passed since the efficacy of postoperative DS chemotherapy has been demonstrated, and further follow-ups are needed. We will continue to analyze and evaluate in the future. Patients who discontinued chemotherapy due to recurrence were excluded from this study to analyze tolerability, and the site of recurrence was not analyzed.

In conclusion, postoperative PS and weight loss rate were independent predictors of DS chemotherapy tolerability after curative surgery for gastric cancer. Efforts to maintain and restore these factors in the perioperative period may contribute to improved tolerability and outcomes for DS chemotherapy.

## Data Availability

The datasets generated during the study are available from the corresponding author on reasonable request. The data is not publicly available due to patient privacy and General Data Protection Regulation.
